# Health status of adults with Short Stature: A comparison with the normal population and one well-known chronic disease (Rheumatoid Arthritis)

**DOI:** 10.1186/1750-1172-2-10

**Published:** 2007-02-27

**Authors:** Heidi Johansen, Inger-Lise Andresen, Eva E Naess, Kare Birger Hagen

**Affiliations:** 1 Sunnaas Rehabilitation Hospital, TRS Resource Centre for Rare Disorders, 1450 Nesoddtangen, Norway; 2 Frambu National Centre for Rare Disorders, 1404 Siggerud, Norway; 3 National Resource Centre for Rehabilitation in Rheumatology, Department of Rheumatology, Diakonhjemmet Hospital, 0370 Oslo, Norway

## Abstract

**Background:**

To examine the subjective health status of adults with short stature (ShSt) and compare with the general population (GP) and one well-known chronic disease, rheumatoid artritis (RA). In addition, to explore the association between age, gender, height, educational level and different aspects of health status of adults with short stature.

**Methods:**

A questionnaire was mailed to 72 subjects with short stature registered in the database of a Norwegian resource centre for rare disorders, response rate 61% (n = 44, age 16–61). Health status was assessed with SF-36 version 2. Comparison was done with age and gender matched samples from the general population in Norway (n = 264) and from subjects with RA (n = 88).

**Results:**

The ShSt sample reported statistically significant impaired health status in all SF-36 subscales compared with the GP sample, most in the physical functioning, Mean Difference (MD) 34 (95% Confidence Interval (CI) 25–44). The ShSt reported poorer health status in mental health, MD 11 (95% CI 4–18) and social functioning, MD 11 (95% CI 2–20) but better in role physical MD 13 (95% CI 1–25) than the RA sample. On the other subscales there were minor difference between the ShSt and the RA sample. Within the short stature group there was a significant association between age and all SF-36 physical subcales, height was significantly associated with physical functioning while level of education was significantly associated with mental health.

**Conclusion:**

People with short stature reported impaired health status in all SF-36 subscales indicating that they have health problems that influence their daily living. Health status seems to decline with increasing age, and earlier than in the general population.

## Background

People with short stature are a heterogenic group with very different clinical expressions due to many different diagnoses with different genetic origins [1]. The literature uses different definitions of short stature: 3SD, 2.5 percentile or 3th percentile below mean height [2-4]. Several medical conditions cause short stature, i.e. skeletal dysplasias, metabolic diseases, growth hormone deficit, chronic diseases like kidney or heart failure and chromosomal disorders. In some cases no cause can be found (idiopathic short stature). The medical literature describes 200–250 different skeletal dysplasias with distinct medical problems. Many need highly specialised medical services [3,5-8].

Short stature caused by skeletal dysplasia is a rare condition; prevalence at birth is estimated to 1/4000. For achondroplasia, the most well known skeletal dysplasia, the prevalence is estimated to 1/15 000–1/40 000 live births [7].

Persons with short stature encounter several challenges in daily life. Many have serious medical problems and their short and often disproportionate stature make life strenuous in an average sized society. The fact that their appearances make them extremely visible in society puts an additional strain to their lives [9,10].

Most studies conducted with people of short stature describe different medical problems and their management [11-18]. The consequences for daily life have been addressed in only a few studies and with different methods. Two Finnish studies [19,20] describe the consequences of short stature for health and quality of life. American studies including only subjects with achondroplasia have described quality of life using different methods [21,22].

To our knowledge, no studies of people with short stature have measured several dimensions of health status with modern methods, or compared health status of people with short stature with other chronic diseases.

In this study the subjective health status of a group of adults with short stature (ShSt) was examined and compared with the health status of the general population (GP) and with that of one other known chronic disease, Rheumatoid Arthritis (RA). In addition the association between age, gender, height, educational level and health status of adults with short stature was explored.

## Methods

The study was designed as a cross-sectional postal survey.

TRS is a national resource centre for rare disorders in Norway. Those who seek the services of TRS are included in a databased register. In October 2004 a questionnaire was mailed to all persons above 16 years of age (72 persons) with skeletal dysplasias (osteogenesis imperfecta excluded) or short stature without known diagnosis. The questionnaires were returned anonymously.

To be able to compare the SF-36 results from the study group with other groups, two comparison groups were established retrospectively: One group from a Norwegian population study and one group from a register of a well known chronic disease, Rheumatoid Arthritis.

SF-36 (version 1) data from the general population was collected by Norwegian Social Science Data Service in "Level of Living 2002" ("Samordnet levekårsundersøkelse"). A dataset from that study was made available to the present study. From this dataset a comparison group was drawn, individually matched on age and gender (6 persons in the general population for each person in the short stature sample).

Diakonhjemmet Hospital, Oslo, established in 1994 Oslo Rheumatoid Arthritis Register (RA-register) [23]. Inclusion criteria in this register are a diagnosis of RA and a residential address in Oslo. Data in the RA-register have been collected through postal surveys, including the SF 36 (version 1) questionnaire. A comparison group was drawn from data collected in 2004. There were very few persons below 20 years; this made individual matching on age impossible. Group matching was done up to 40 years, while individual matching on age and gender was done for ages above 40 (2 persons from the RA-register for each person in the short stature sample).

The survey instruments included demographic data, disease-specific questions (health problems, medical treatment and the use of welfare services) and the generic health status instrument MOS SF-36v2. SF-36 is widely used for measuring subjective health status [24,25]. It consists of eight subscales (four mental and four physical): mental health (mh), role functioning emotional (re), social functioning (sf), vitality (vt) and general health (gh), bodily pain (bp), physical functioning (pf), role functioning physical (rp). SF36 exists in two versions; SF-36 and SF-36v2. The last version corrects deficiencies in the original version (especially in the two role subscales) and is calibrated so that the results from the two versions can be compared [24]. All subscales of the SF-36 have 0–100 scales, 100 is best health status score. The mean score and variance vary considerably between the subscales in normative data.

The data were entered into SPSS 13.0 for Windows for descriptive and statistical analyses. SF-36 was scored according to Health survey manual & interpretation guide [25]. Independent sample t-tests were used to compare the short stature group with the general population and the Rheumatoid Arthritis groups. The sample size of both the study group and the comparison groups (n>30) allow use of independent sample t-test [26]. When checked for normality, all variables in the study group were found to be distributed normally. In the comparison groups the distribution was however somewhat skewed (especially in the role subscales). Non-parametric tests (Mann-Whitney test) were therefore also used.

Univariate and multiple linear regressions were used to assess the influence of age, gender, educational level and height on the SF-36 scores for the short stature group. Linear regression was also used to assess the influence of age and gender on SF-36 scores for the general population. All models were thoroughly checked for violations from the linear model assumptions.

National Committee for Research Ethics in Norway approved the study March 2004

## Results

The questionnaire was mailed to 72 persons with short stature, mean age 35 years, (range 16–71), 67% females. After one reminder 44 persons (61%) responded. The mean age of the respondents was 36,4 years (range 16–61), 32 (73%) were females. The characteristics of the short stature sample are shown in table 1.

**Table 1:** Characteristisics of the short stature sample (N = 44)

Characteristics	n (%)	Mean (SD)	Range
Age		36.4 (13)	16 – 61
Weight		55,5 (13)	30 – 85
Height		134 (13)	94 – 156
			
Male	12 (27)		
Married	16 (36)		
Parenthood	13 (29)		
Mobility: use wheelchair	13 (29)		
Formal education >12 years	16 (36)		
Employment status			
Under education	15 (34)		
Employed	19 (43)		
Disability pension	10 (23)		
Diagnosis reported			
Achondroplasia	19 (43)		
Hypochondroplasia	2 (5)		
Pseudoachondroplasia	3 (7)		
Spondyloepiphyseal dysplasia	3 (7)		
Other skeletal dysplasias*	9 (20)		
No diagnosis reported	8 (18)		

* chondrodystrophia (1), diastrophic dysplasia (1), dyschondrosteose (2), dysplasia epiphysialis multiplex (2), metaphysial chondrodysplasia (1), metatropic dysplasia (1), spondylo meta physical dysplasi Kozlowskys type (1)

Almost all the respondents had various skeletal dysplasias; achondroplasia being the most frequent diagnosis (43%), while 18% reported that they did not know any diagnosis for their short stature. This latter group was analysed for height (mean height 135,6 cm; range 127–146), which is not significantly different from the rest of the study sample.

The short stature sample (ShSt) reported impaired health status compared to the general population (GP). Statistical significance was reached in all SF-36 subscales. The difference between the GP and ShSt was particularly prominent in the physical subscales: general health, bodily pain, physical functioning and role physical, (27–34 point difference on a 0–100 scale) (table 2). The results applied to both genders (data not shown).

**Table 2:** Mean (Standard Deviation) SF-36 scores for the ShSt, the GP and the RA samples. Differences (95% confidence interval) between GP and ShSt and between RA and ShSt.

SF-36 subscales	Mean(SD) SF-36 scores			Differences GP-ShSt		Differences RA-ShSt	
			
	ShSt n = 44	GP n = 264	RA n = 88	Mean diff (95%CI)	p-value	Mean diff (95%CI)	p-value
Mental health	65(22)	81(14)	76(15)	15 (9 to 22)	<0.001	11 (4 to 18)	0.003
Role emotional	74(31)	90(26)	77(37)	16 (6 to 26)	0.002	3 (-9 to 15)	0.663
Social functioning	65(26)	90(17)	76(24)	24 (16 to 33)	<0.001	11 (2 to 20)	0.021
Vitality	41(16)	64(17)	47(20)	23 (18 to 28)	<0.001	7 (0 to 13)	0.040
General health	51(23)	82(17)	54(24)	32 (24 to 39)	<0.001	4 (-5 to 12)	0.403
Bodily pain	47(27)	79(24)	47(19)	31 (23 to 40)	<0.001	-1 (-10 to 8)	0.884
Physical functioning	58(31)	93(13)	66(25)	34 (25 to 44)	<0.001	8 (-3 to 18)	0.160
Role physical	60(30)	88(27)	47(40)	27 (17 to 37)	<0.001	-13 (-25 to -1)	0.038

ShSt: short stature, GP: general population, RA: rheumatoid arthritis

Comparison between the ShSt and Rheumatoid Arthritis (RA) samples showed small differences in health status in most subscales. Persons with ShSt had however higher scores in the physical role subscale and lower scores in the mental health, social functioning and vitality subscales compared to the RA sample. These differences were statistically significant (table 2). All the differences that were statistically significant when t-tests were used were also statistically significant when non-parametric tests were used (results not shown).

Within the ShSt group, the results showed large variation in all subscales, indicating that some had scores on or above mean scores for the general population. For instance, in the physical functioning subscale, 35% scored 75 and higher, indicating good physical functioning, and 22% scored 25 or less, indicating poor physical functioning.

People with achondroplasia reported higher scores than people with the other diagnoses in the subscales bodily pain and physical functioning. Comparisons of health status showed minor differences in the other subscales, none of the differences were statistically significant (data not shown).

Table 3 shows the results of univariate and multivariate regression analyses for the ShSt group, with age, gender, height and level of education as independent variables and the eight subscales in the SF-36 as dependent variables. There was a significant association between age and all physical subscales (general health, bodily pain, physical functioning and role physical) and with the vitality subscale showing lower scores with higher age. On the physical functioning subscale there was also a significant association with height showing lower scores with lower height. Level of education is the best predictor of health status in the mental health and role emotional subscales, with higher scores for those with more than 12 years of education. In all subscales men had higher scores than women, but the associations were not statistically significant.

**Table 3:** Regression coefficients (95% confidence interval) and p-value for age, gender, height and level of education related to SF36 dimensions for the short stature sample (n = 44).

SF-36 subscales	Variables	Univariat regression		Multiple regression	
			
		Coefficient (95%CI)	p-value	Coefficient (95%CI)	p-value
Mental health	Age	-0.1 (-0.6 to 0.5)	0.817	-0.1 (-0.7 to 0.4)	0.572
	Gender	-9.5 (-24.3 to 5.2)	0.200	-5.3 (-20.9 to 10.3)	0.497
	Height	0.2 (-0.3 to 0.7)	0.451	-0,1 (-0.6 to 0.5)	0.795
	Level of education	17.1 (4.2 to 30.0)	0.010	18.1 (3.7 to 32.5)	0.015
					
Role emotional	Age	-0.5 (-1.2 to 0.2)	0.153	0.6 (-1.4 to 0.1)	0.097
	Gender	-12.1 (-32.9 to 8.8)	0.250	-7.1 (-29.3 to 15.1)	0.523
	Height	0.2 (-0.5 to 1.0)	0.567	-0.1 (-0.9 to 0.7)	0.727
	Level of education	17.3 (-1.5 to 36.2)	0.071	21.0 (0.6 to 41.5)	0.044
					
Social functioning	Age	-0.4 (-1.0 to 0.2)	0.219	0.3 (-1.0 to 0.3)	0.344
	Gender	-14.7 (-32.3 to 2.8)	0.098	-11.0 (-30.6 to 8.7)	0.266
	Height	0.3 (-0.3 to 1.0)	0.322	0.2 (-0.5 to 0.9)	0.623
	Level of education	1.7 (-15.1 to 18.5)	0.842	0.9 (-17.2 to 19.0)	0.921
					
Vitality	Age	-0.5 (-0.8 to -0.1)	0.010	-0.5 (-0.8 to 0.1)	0.020
	Gender	-6.6 (-17.6 to 4.5)	0.236	-3.8 (-15.3 to 7.7)	0.509
	Height	0.1 (-0.2 to 0.5)	0.461	0.0 (-0.4 to 0.4)	0.874
	Level of education	1.9 (-8.4 to 12.3)	0.708	3.9 (-6.7 to 14.5)	0.460
					
General Health	Age	-0.6 (-1.1 to 0.1)	0.020	-0.6 (-1.2 to -0.0)	0.043
	Gender	-2.8 (-18.8 to 13.3)	0.729	1.0 (-16.1 to 18.2)	0.904
	Height	0.2 (-0.4 to 0.8)	0.460	0.2 (-0.4 to 0.8)	0.588
	Level of education	0.6 (-15.5 to 14.3)	0.936	1.8 (-13.9 to 17.6)	0.814
					
Bodily Pain	Age	-0.8 (-1.4 to -0.3)	0.005	-0.9 (-1.5 to -0.3)	0.007
	Gender	-6.1 (-24.4 to 12.2)	0.507	-6.3 (-25.4 to 12.8)	0.507
	Height	0.1 (-0.5 to 0.8)	0.653	0.0 (-0.6 to 0.7)	0.917
	Level of education	-2.8 (-20.0 to 14.2)	0.737	-0.3 (-17.9 to 17.2)	0.971
					
Physical functioning	Age	-1.0 (-1.7 to -0.4)	0.004	-0.9 (-1.5 to -0.3)	0.005
	Gender	-15.4 (-36.0 to 5.2)	0.139	-3.6 (-22.1 to 15.0)	0.699
	Height	1.2 (0.5 to 1.8)	0.001	1.1 (0.4 to 1,7)	0.002
	Level of education	5.9 (-13.5 to 25.4)	0.541	2.2 (-14.9 to 19.3)	0.795
					
Role Physical	Age	-1.1 (-1.7 to -0.4)	0.001	-1.1 (-1.7 to -0.4)	0.002
	Gender	-13,8 (-34.2 to 6.6)	0.180	-5.4 (-25.2 to 14.3)	0.579
	Height	0.7 (0.0 to 1.4)	0.050	0.5 (-0.2 to 1.2)	0.161
	Level of education	6.5 (-12.7 to 25.6)	0.499	7.7 (-10.5 to 25.9)	0.396

Coding of categorical variables: male = 1, female = 2; level of education < 12 years = 1, level of education >12 years = 2. Age and height were treated as continuous.

Regression analyses for the GP group with age and gender as independent variables showed few significant associations with most SF-36 subscales. Higher age was associated with higher SF-36 scores on mental health and vitality and lower score on bodily pain (data not shown).

## Discussion

This study highlights the fact that living with short stature affects people across all major dimensions of health as measured with SF-36 and affects most in the physical subscales. Compared with a sample from the general population matched on gender and age, men and women with short stature report significantly impaired health status. The low scores in the physical subscales are in accordance with an American study of people with achondroplasia that used SF-36 [21]. They found significant lower "Physical Component Summary" (a weighted score from the four physical subscales) for people with achondroplasia than for the general population, but they found no differences on the "Mental Component Summary". Another study used a Ferran and Powers Quality of Life Index, and found lower scores for people with achondroplasia compared with their unaffected first-degree relatives [22]. The results of the present study are also in accordance with results from two Finnish studies [19,20]. Those studies mainly described other short stature diagnoses and used other health status questionnaires, but the main results showed the same tendency.

When compared with people with rheumatoid arthritis, people with short stature report nearly equal health status in some subscales; better health status in role physical and lower health status in mental health and social functioning subscales. Rheumatoid Arthritis is a well-known chronic, autoimmune, inflammatory disease that causes pain and gradual physical disability. Short stature and skeletal dysplasias are less known and other people may fail to understand the health challenges and problems that people with these conditions meet. A comparison with a well-known group was done to clarify the extent of these health challenges.

It is well documented that the physical health status decreases with increasing age in the general population [27]. Even though the mean age in this study group is quite low (36,4 years), age is still the most important predictor of health status in all the physical subscales for people with short stature. This was not the case in the age-matched comparison group from the general population. This indicates that the decline in health status starts earlier in people with short stature than in the general population. This corresponds to the findings of Mahomed [21] where the scores in the physical domain declined significantly after the fourth decade of life for people with achondroplasia. Literature describes premature degenerative arthritis as a common problem with increasing age in some of the skeletal dysplasia diagnoses, i.e. pseudoachondroplasia, multiple epiphyseal dysplasia [4,5,7]. The same applies for neurological pathology in other skeletal dysplasia diagnoses, i.e. achondroplasia, hypocondroplasia and spondyloepiphyseal dysplasia [4,5,7]. The pain and physical disability associated with these pathologies may contribute to the early decrease of health status in the short stature sample. Studies on other disabilities also show that they have increasing physical problems relatively early in life [28,29].

The level of education is the best predictor on the mental health and role emotional dimensions which is in accordance with findings in a study of health status of people with ankylosing spondylitis [29].

Gollust et al [22] reported a statistically significant association between gender and the total QOL score (and the subscales "health and functioning", "psychological and spiritual") with higher scores for men than for women. In the present study group, the same relationship between gender and all SF-36 subscales was found, but the associations did not reach statistical significance.

The present findings must be interpreted within the limitations of the study. Only a part of the Norwegian short stature population is registered at TRS Resource Centre for Rare Disorders and only 61 % of the eligible persons answered the questionnaire. As we have no information of those not registered at TRS, there may be a selection bias. The data that were used to compare the groups contained data from different studies and also different versions of SF 36 were used. This makes comparisons of the role functioning subscales somewhat uncertain. The level of education has in other studies proved to influence health status [27,29]. The lack of information on the level of education in the comparison groups may therefore contribute to a bias in the results. However, aggregated data from Statistics Norway [30] indicate that the education level of the ShSt sample does not differ much from the general population of Norway (30% >12 years of education). It is reasonable to assume that the GP sample has a similar level of education as the population at large, and it is therefore not likely that the differences in SF-36 scores can solely be explained by differences in education level.

The number of persons in this survey is small, and in particular, there are few men. Generalizations from these results must therefore be made with caution. Finally, the study sample is very heterogeneous in relation to the different diagnoses that are included. The results showed, however, no statistically significant differences between the achondroplasia group and the other diagnoses put together. This corresponds with the results from Apajasalo et al [19] who compared three different diagnoses; achondroplasia, cartilage-hair- nail hypoplasia and diastrophic dysplasia and found only small differences in health status between the groups.

## Conclusion

To our knowledge this is the first study in Norway that describes health status in people with short stature by a standardized questionnaire. The results may help to increase the understanding of the special challenges this group meets in every day life and thereby be useful to health personnel in giving services to people with short stature. The findings indicate that the everyday lives of persons with short stature are often strenuous. It is important that health care professionals and other professionals understand that living in an average sized world when you are short, may strain you both physically and mentally. Their short stature, their visible appearance and their medical complications may affect their health status. It is, however, important to bear in mind that the large variations in the SF-36 scores also indicate that not all people with short stature have impaired health status, some of them are in good health, both physically and mentally.

These results may throw light on some aspects of the lives of people with short stature. More studies are needed to investigate into the problems with early aging and the possibility to take actions so that people with short stature may live less strenuous lives.

## Competing interests

The author(s) declare that they have no competing interests.

## Authors' contributions

HJ participated in the design of the study, the data collection, the statistical analysis, interpretation of the results and drafted the manuscript. ILA participated in the data collection, the statistical analysis, interpretation of the results and drafted the manuscript. EEN participated in the design of the study, interpretation of the results and drafted the manuscript. KBH supervised the study process from the start and revised the manuscript critically. All authors read and approved the final manuscript.
